# Therapeutic Potential of Local and Systemic Adipose-Derived Mesenchymal Stem Cell Injections in a Rat Model of Experimental Periodontitis: Implications for Cardiac Function

**DOI:** 10.3390/ijms26093984

**Published:** 2025-04-23

**Authors:** Asmaa Elhaieg, Ahmed Farag, Sai Koung Ngeun, Masahiro Kaneda, Aimi Yokoi, Ahmed S. Mandour, Ryou Tanaka

**Affiliations:** 1 Veterinary Teaching Hospital, Tokyo University of Agriculture and Technology, Tokyo 183-8509, Japan; ahmedfarag9331@gmail.com (A.F.);; 2 Department of Surgery, Anesthesiology, and Radiology, Faculty of Veterinary Medicine, Zagazig University, Zagazig 44519, Egypt; 3 Department of Neurophysiology, National Center of Neurology and Psychiatry, Tokyo 187-8551, Japan; 4 Laboratory of Veterinary Anatomy, Division of Animal Life Science, Tokyo University of Agriculture and Technology, Tokyo 183-8509, Japan; 5 Department of Animal Medicine (Internal Medicine), Faculty of Veterinary Medicine, Suez Canal University, Ismailia 41522, Egypt

**Keywords:** adipose-derived mesenchymal stem cells, periodontitis, cardiac dysfunction, myocardial fibrosis, H&E staining, Masson’s trichrome staining, radiography, echocardiography

## Abstract

Periodontitis is a common inflammatory disease that not only damages periodontal tissues but also induces systemic effects, including cardiac dysfunction. Mesenchymal stem cells (MSCs) offer regenerative potential due to their ability to differentiate, modulate immune responses, and secrete anti-inflammatory factors. However, the relative efficacy of local versus systemic MSC administration remains unclear. This study evaluated the therapeutic effects of adipose-derived MSCs (AD-MSCs) in a rat model of experimental periodontitis, comparing local and systemic administration. AD-MSCs were characterized based on morphology, surface marker expression, and differentiation potential. Ligature-induced periodontitis was established over 60 days, after which AD-MSCs (1 × 10^6^ cells) were administered either supraperiosteally (local group) or intravenously (systemic group). Periodontal regeneration was assessed through clinical, radiographic, and histopathological analyses, while cardiac function was evaluated using echocardiography and histopathological examinations. Results demonstrated that local AD-MSC administration provided superior therapeutic benefits compared to systemic delivery. Locally administered cells significantly enhanced bone regeneration, reduced inflammation, and improved periodontal tissue architecture. In contrast, systemic administration offered moderate benefits but was less effective in restoring periodontal integrity. Similarly, in the heart, local treatment resulted in greater improvements in systolic function, as indicated by enhanced ejection fraction and fractional shortening, along with reduced myocardial fibrosis. Although systemic administration also provided cardioprotective effects, diastolic dysfunction persisted in both treatment groups. In conclusion, local AD-MSC administration proved more effective in regenerating periodontal tissues and mitigating cardiac dysfunction, highlighting its potential as an optimized therapeutic strategy for periodontitis and its systemic complications.

## 1. Introduction

Periodontitis is a widespread condition, affecting up to 90% of the global population. It primarily impacts the oral and maxillofacial region and is characterized by the progressive destruction of tooth-supporting structures, including the alveolar bone, periodontal ligament (PDL), and root cementum. If left untreated, it leads to ongoing periodontal attachment loss and bone deterioration, ultimately causing premature tooth loss [1]. This condition presents a significant challenge for dental professionals, patients, and public dental healthcare systems. Notably, traditional therapeutic approaches, whether surgical or non-surgical, fail to fully regenerate the damaged periodontal support structures resulting from the disease [2]. The substantial burden of periodontitis, combined with its adverse effects on patients’ quality of life, underscores the necessity for more effective treatment strategies [3,4].

Mounting evidence confirms periodontitis as an independent risk factor for cardiovascular disease (CVD), with shared pathological mechanisms now well characterized. Chronic periodontal inflammation triggers systemic release of pro-inflammatory cytokines (IL-1β, IL-6, TNF-α) that accelerate endothelial dysfunction and promote atherosclerotic plaque instability [5]. This inflammatory spillover is exacerbated by periodontal pathogens like *Porphyromonas gingivalis*, which activate vascular TLR-2/4 receptors, stimulating NF-κB pathways that amplify systemic inflammation [6]. Notably, *P. gingivalis* evades immune surveillance and disseminates via blood and lymphatic circulation, enabling its colonization of arterial walls and initiation of local vascular inflammation. Moreover, it disrupts lipid metabolism and promotes systemic immune activation, accelerating atherosclerotic progression [7]. Clinical studies have detected oral pathogen DNA in human atheromatous plaques, with *P. gingivalis* enhancing atherosclerotic plaque formation by upregulating LOX-1 expression, thereby promoting endothelial activation, monocyte adhesion, and the release of inflammatory mediators in the vascular wall [8,9].

Beyond atherosclerosis, periodontitis has been suggested as a potential risk factor for cardiovascular conditions, including atrial fibrillation (AF) and acute myocardial infarction (AMI). A study on dogs with induced periodontitis showed that inflammation from periodontal disease led to structural and electrophysiological changes in the atrium, facilitating AF development [10]. Similarly, in a community-based cohort, men with a history of periodontitis exhibited higher myocardial fibrosis, linking periodontal disease with cardiovascular risks [11]. Heat shock proteins (HSP60) are implicated in inflammation at sites like atherosclerotic plaques, further supporting the inflammatory pathway linking periodontitis to cardiovascular diseases [12]. Additionally, a study on AMI patients found that those with periodontitis had elevated oxidative stress markers, suggesting a common pathogenic mechanism between the two conditions [13]. These findings underscore the potential role of periodontitis as both a risk factor and a contributor to the progression of cardiovascular diseases.

MSCs are multipotent adult progenitor cells capable of differentiating into various mesenchymal lineages, including cartilage, adipose tissue, bone marrow stroma, and bone [14,15,16]. Their ability to differentiate into these specialized cell types has been the basis for numerous therapeutic applications, such as enhancing bone regeneration [17] and repairing cartilage defects [17,18]. Beyond their differentiation potential, MSCs have demonstrated therapeutic benefits in various repair processes by secreting trophic factors [19]. These factors facilitate tissue repair by promoting vascularization, preventing cell death, and modulating immune responses, making MSCs a versatile tool in regenerative medicine [20,21].

MSC-based tissue regeneration holds promise as a potential therapeutic approach for periodontal tissue repair. However, the optimal method of exogenous MSC application, whether local or systemic, remains uncertain [22]. Bioluminescence imaging studies have shown that no detectable signal was observed in the periodontal lesion of mice injected with MSCs via the tail vein. Instead, strong signals were detected in the lungs and liver immediately after injection, suggesting that systemically administered MSCs became trapped in these organs. Despite the presence of periodontal injury, MSCs did not migrate in response to inflammatory mediators or home to the wound site after initial lung entrapment [23,24,25]. These findings indicate that systemic administration of MSCs may not be the preferred approach for periodontitis models. Instead, localized delivery of stem cells may enhance cell engraftment and improve therapeutic outcomes.

Current clinical management of periodontitis includes mechanical debridement (scaling and root planing), adjunctive antibiotics, and regenerative approaches such as guided tissue regeneration (GTR) or enamel matrix derivatives (EMDs) [26,27]. The latest classification system by the American Academy of Periodontology and European Federation of Periodontology provides a standardized diagnostic framework [26], while recent studies indicate comparable early wound healing outcomes for GTR and EMD, without clear superiority of one over the other [27].

However, these approaches often fail to fully restore the periodontal architecture, particularly in advanced cases, highlighting the need for innovative therapies [28]. MSCs have emerged as a promising strategy, with human trials demonstrating their safety and efficacy in periodontal regeneration. For instance, a randomized controlled trial reported that local injection of dental pulp stem cells (DPSCs, a MSC type with similar regenerative properties to BM-MSCs) significantly improved clinical attachment levels and radiographic bone fill in periodontitis patients over 12 months [29]. Similarly, MSCs loaded onto a composite scaffold of demineralized bone mineral and calcium sulphate (Osteoset) achieved 34.5% bone regeneration bridging the cleft in one unilateral alveolar cleft case, and 25.6% bone integrity restoration in another, with no reported complications at 4 months [30].

Despite these advances, challenges remain, including standardization of MSC sources, optimal delivery methods, and long-term stability of regenerated tissues [31]. Additionally, the comparative efficacy of local versus systemic administration in treating periodontitis and its systemic complications, particularly cardiac dysfunction, remains poorly understood. Furthermore, the systemic benefits of AD-MSCs, including their cardioprotective effects, have not been thoroughly investigated. We hypothesized that local administration of AD-MSCs would be more effective than systemic delivery in promoting periodontal regeneration and mitigating periodontitis-associated cardiac dysfunction due to enhanced cell retention and targeted action at the injury site.

This study aims to evaluate the therapeutic efficacy of AD-MSCs administered locally and systemically in a rat model of ligature-induced periodontitis, with a focus on their effects on periodontal regeneration and periodontitis-associated cardiac dysfunction. By comparing these approaches, this study seeks to optimize MSC delivery strategies and maximize their regenerative and cardioprotective potential.

## 2. Results

### 2.1. Characterization of Rat AD-MSCs

#### 2.1.1. Morphology

**Figure 1:**
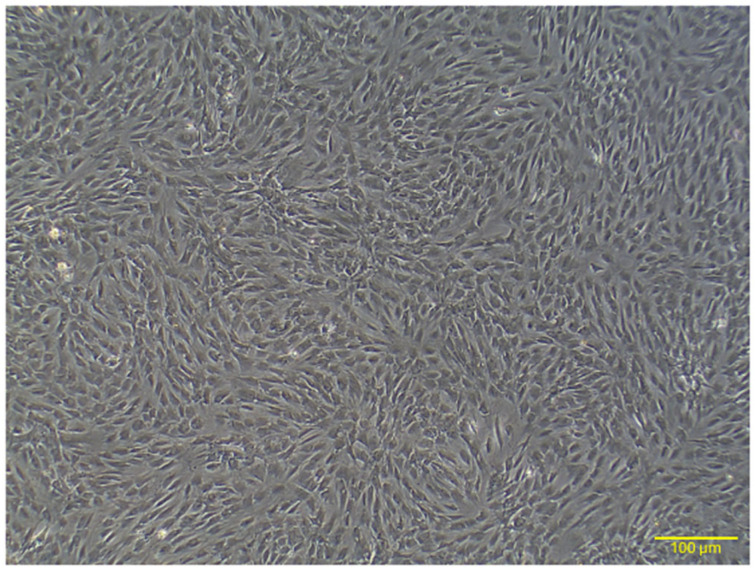
Representative image illustrating the morphology of rat AD-MSCs. At passage 4, the AD-MSCs displayed a spindle-shaped, fibroblast-like structure. Scale bar: 100 μm.

At passage 4, the cultured adipose tissue-derived mesenchymal stem cells (AD-MSCs) displayed a fibroblast-like morphology, as shown in Figure 1. The cells adhered to the culture surface, exhibiting a spindle-shaped and elongated structure characteristic of mesenchymal stem cells.

#### 2.1.2. Flow Cytometry Analysis

**Figure 2:**
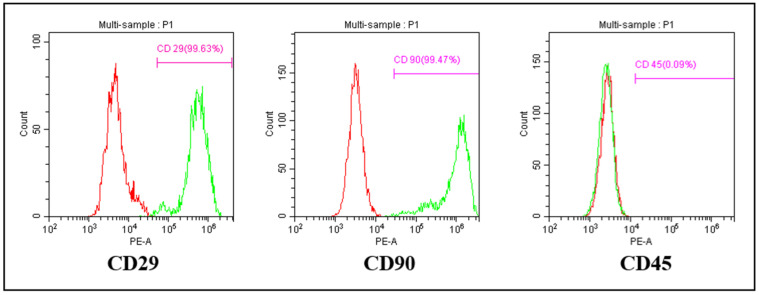
Phenotypic characterization of AD-MSCs. Flow cytometric analysis shows that the cultured AD-MSCs express CD29 and CD90, while lacking CD45 expression.

Flow cytometric analysis revealed that the AD-MSCs were positive for CD29 and CD90, which are characteristic surface markers of mesenchymal stem cells. In contrast, the cells were negative for CD45, a hematopoietic marker, confirming the mesenchymal origin of the cultured cells (Figure 2).

#### 2.1.3. Multilineage Differentiation

**Figure 3:**
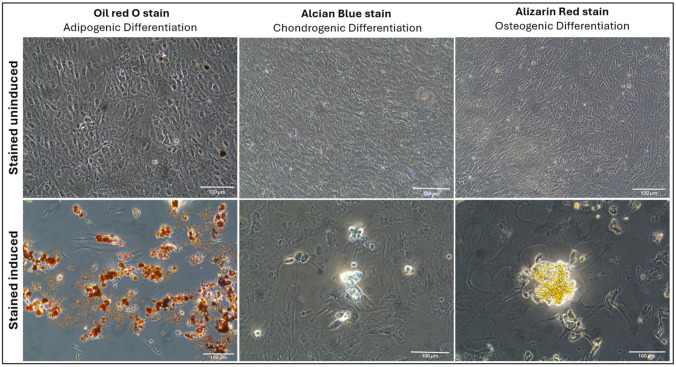
Multilineage differentiation of rat AD-MSCs. Adipogenesis was confirmed by Oil Red O staining, chondrogenesis by Alcian Blue staining, and osteogenesis by Alizarin Red staining. Scale bar: 100 μm.

To evaluate the multilineage differentiation potential of passage-four AD-MSCs, the cells were induced to differentiate into adipogenic, chondrogenic, and osteogenic lineages. Adipogenic differentiation was validated through Oil Red O staining, which demonstrated the presence of lipid droplets, confirming the successful differentiation of AD-MSCs into adipocytes. Chondrogenic differentiation was verified by Alcian Blue staining, which detected sulfated proteoglycans, confirming the formation of cartilage-like structures. Osteogenic differentiation was demonstrated by an increase in Alizarin Red staining, highlighting calcium deposits within osteoblasts, indicative of osteogenic maturation (Figure 3). These findings collectively confirm that the isolated AD-MSCs retain the capacity for multilineage differentiation, supporting their potential for regenerative applications.

### 2.2. Bone Loss Assessment

**Figure 4:**
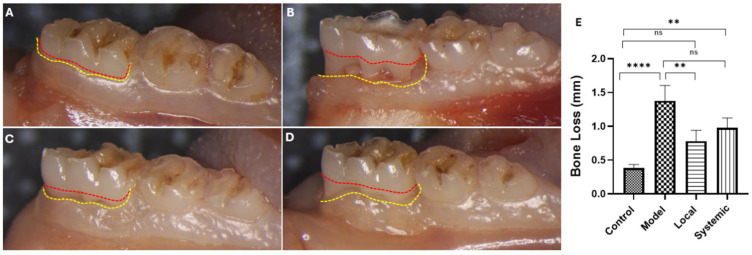
Clinical assessment of alveolar bone loss in experimental groups. (**A**–**D**) Gross images of molar regions: (**A**) control group, (**B**) model group, (**C**) local AD-MSC-treated group, and (**D**) systemic AD-MSC-treated group. Red and yellow dashed lines indicate the cemento-enamel junction (CEJ) and alveolar bone crest (ABC), respectively. (**E**) Quantitative measurement of bone loss (mm) across groups. **** *p* < 0.0001, ** *p* < 0.01, ns = not significant.

The clinical evaluation revealed a significant increase in the distance from the cemento-enamel junction (CEJ) to the alveolar bone crest in the model group compared to the control group (*p* < 0.0001), indicating severe bone loss and root exposure. Local administration of AD-MSCs significantly reduced bone loss compared to the model group (*p* < 0.01), with measurements recovering to levels nearly equivalent to the control group (*p* > 0.05). In contrast, systemic AD-MSC administration showed limited effects on alveolar bone repair. Although a reduction in bone loss was observed, it was not statistically significant compared to the model group and remained significantly higher than the control group (*p* < 0.01) (Figure 4).

### 2.3. Alveolar Bone Radiographic Analyses

**Figure 5:**
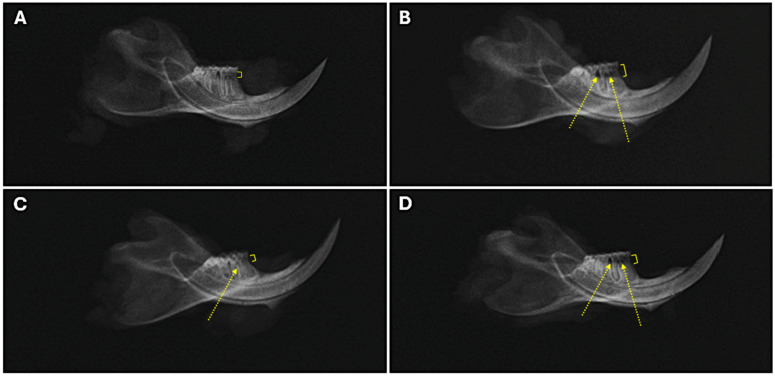
Radiographic analysis of alveolar bone levels. (**A**–**D**) Representative radiographs of mandibular regions: (**A**) control group, (**B**) model group, (**C**) local AD-MSC-treated group, and (**D**) systemic AD-MSC-treated group. Brackets indicate bone loss measured as the distance from the cemento-enamel junction (CEJ) to the alveolar bone crest. Arrows highlight bone loss in the interproximal and interradicular regions.

Radiographic analysis of alveolar bone destruction (Figure 5) further supported the clinical findings. Quantitative analysis revealed significantly greater bone loss and reduced bone support in the model group compared to controls (*p* < 0.001; Table 1). The local treatment group showed bone loss and bone support values similar to those of the control group (*p* > 0.05), suggesting enhanced bone formation in periodontal defects. In contrast, the systemic treatment group exhibited significant bone loss compared to controls (*p* = 0.032) but showed a trend toward significant (*p* = 0.07) partial restoration of bone support. These findings highlight the superior efficacy of local AD-MSC administration in mitigating bone destruction associated with periodontal disease.

### 2.4. Histopathological Analysis of Mandibular Tissues

**Figure 6:**
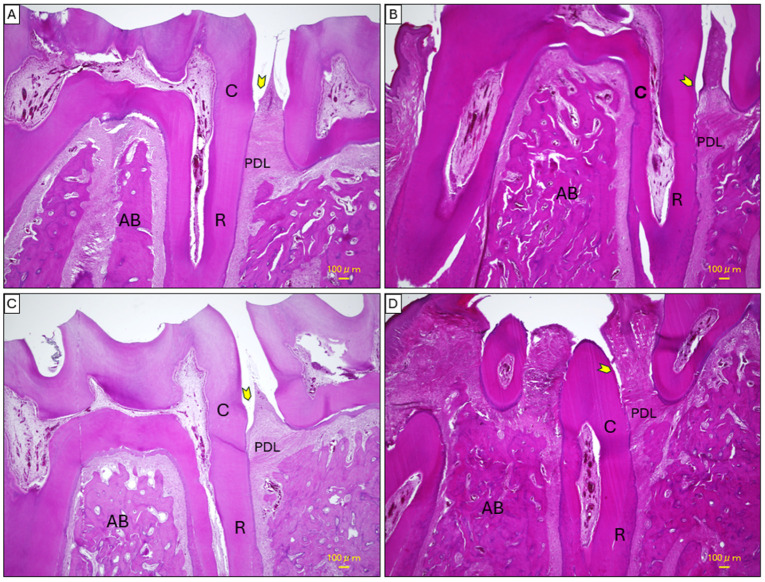
Histopathological analysis of mandibular first molar tissues using H&E staining. (**A**) Control group showing intact tissue architecture with no inflammation or bone loss. (**B**) Model group displaying severe apical migration of the junctional epithelium and disorganized periodontal ligament (PDL). (**C**) Local treatment group showing partial restoration of tissue structure and reduced inflammation. (**D**) Systemic treatment group showing moderate recovery with less inflammation and structural improvement compared to the local group. Arrowheads indicate the position of the junctional epithelium in all panels. Scale bar: 100 μm. Abbreviations: C: cementum, R: tooth root, AB: alveolar bone, PDL: periodontal ligament.

Regarding H&E staining, in the control group (Figure 6A), the mandibular tissues displayed a well-preserved architecture with healthy interproximal areas. The junctional epithelium remained close to the cemento-enamel junction (CEJ), and the alveolar bone was intact without signs of inflammation or structural disruption. The periodontal ligament (PDL) appeared properly expanded, and Sharpey’s fibers were observed smoothly integrating into the alveolar bone surface, which was even in contour. In contrast, the model group (Figure 6B) exhibited severe tissue degradation characterized by significant apical migration of the junctional epithelium, disorganization of collagen fibers within the PDL, and pronounced alveolar bone loss. This led to the formation of periodontal pockets, with inflammatory cell infiltration evident around the alveolar bone and the PDL margins. The alveolar bone surface was irregular, and the interradicular bone appeared fragmented into island-like spicules, creating a networked appearance infiltrated by inflammatory cells.

The local treatment group (Figure 6C) demonstrated notable tissue recovery compared to the model group. There was a reduction in apical migration of the junctional epithelium, with improved bone structure and decreased inflammatory cell infiltration. The PDL began regaining its normal structure, appearing less thickened, and interradicular areas showed partial restoration. The systemic treatment group (Figure 6D) showed moderate improvement over the model group but exhibited less recovery compared to the local treatment group. Evidence of healing included reduced cellular infiltration and partially restored tissue organization. However, the periodontal spaces remained thickened, and ongoing remodeling was observed. Apical migration of the junctional epithelium and alveolar bone loss were less pronounced than in the model group, yet tissue integrity was not fully restored.

**Figure 7:**
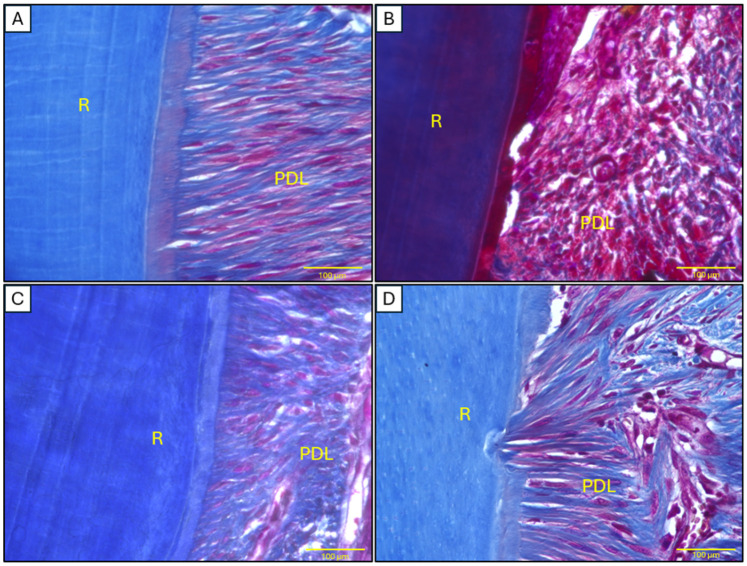
Histopathological analysis of mandibular tissues (periodontal ligament) using Masson’s trichrome staining. (**A**) Control group showing dense, well-organized collagen fibers. (**B**) Model group displaying sparse and disorganized collagen fibers. (**C**) Local treatment group demonstrating improved collagen density and organization. (**D**) Systemic treatment group exhibiting increased collagen deposition but less organization compared to the local group. Scale bar: 100 μm. Abbreviations: R: tooth root, PDL: periodontal ligament.

Masson’s trichrome staining demonstrated distinct patterns of collagen organization across experimental groups (Figure 7). Quantitative analysis revealed significantly higher collagen density in control animals (79.86 ± 2.27%), exhibiting the characteristic dense, well-structured fibers of healthy periodontal ligament tissue. In contrast, the model group showed marked collagen depletion (41.32 ± 1.43%, *p* < 0.001 vs. control), with disorganized fiber architecture and prominent muscle fiber staining indicative of periodontitis-induced structural damage. Local AD-MSC treatment achieved near-complete collagen restoration (70.82 ± 2.22%), showing no statistical difference from controls (*p* > 0.05) while being significantly improved over the model group (*p* = 0.04). Systemic administration produced intermediate results (60.29 ± 1.30%), demonstrating partial but statistically significant recovery compared to controls (*p* = 0.04) yet failing to show significant improvement over the model group (*p* > 0.05), with less organized fiber patterns than local treatment.

### 2.5. Histopathological Analysis of Cardiac Tissues

**Figure 8:**
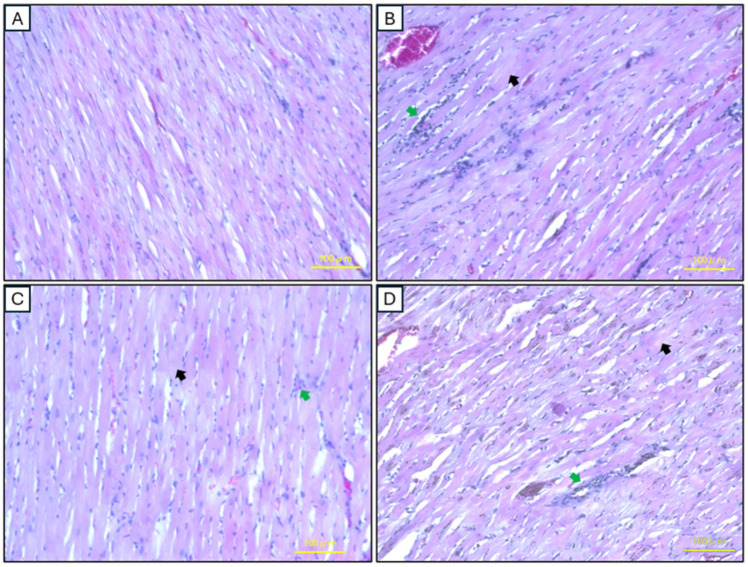
Histopathological analysis of cardiac tissues using Hematoxylin and Eosin (H&E) staining. (**A**) Control group showing normal cardiac morphology with well-organized cardiomyocytes. (**B**) Model group exhibiting inflammatory cell infiltration and cell degeneration. (**C**) Local AD-MSC injection group demonstrating improved cardiac morphology with reduced inflammation and partial restoration of tissue organization. (**D**) Systemic AD-MSC injection group showing reduced inflammation and partial recovery of myocardial architecture (Green arrow: inflammatory cell infiltration; black arrow: degenerated cells). Scale bar: 100 μm.

In the control group, H&E staining reveals normal cardiac morphology. The cardiomyocytes are arranged in longitudinal bundles with centrally located nuclei, displaying a normal myocardial muscle structure and interstitial tissue without evidence of inflammation, edema, or necrosis. In contrast, the model group exhibits significant structural abnormalities, including inflammatory cell infiltration, cell degeneration, and a discernible absence of nucleation. The local AD-MSC injection group demonstrates marked improvement in cardiac morphology, with a noticeable reduction in inflammation, partial restoration of tissue organization, and relatively normalized cardiomyocyte alignment, although mild inflammation persists. Similarly, the systemic AD-MSC injection group shows reduced inflammatory cell infiltration and partial recovery of normal myocardial architecture. However, the improvements in this group are slightly less pronounced compared to the local injection group (Figure 8).

**Figure 9:**
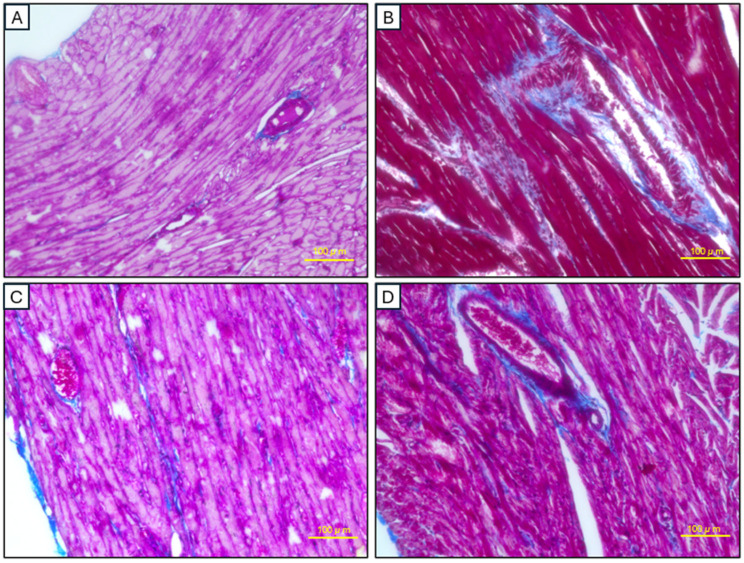
Histopathological analysis of cardiac tissues using Masson’s trichrome staining. (**A**) Control group showing minimal collagen deposition, indicating the absence of fibrosis. (**B**) Model group displaying significant collagen deposition and fibrosis. (**C**) Local AD-MSC injection group exhibiting minimal fibrosis and improved structural integrity of cardiomyocytes. (**D**) Systemic AD-MSC injection group showing reduced fibrosis compared to the model group but with slightly higher collagen deposition than the local injection group. Scale bar: 100 μm.

The extent of myocardial fibrosis was rigorously quantified through histomorphometric analysis of Masson’s trichrome-stained sections (Figure 9). Control myocardium exhibited minimal interstitial collagen deposition (2.6 ± 0.3%), consistent with preserved tissue architecture. Ligature-induced periodontitis triggered profound fibrotic remodeling, with collagen content increasing to 21.04 ± 1.1% (*p* < 0.001 vs. control), accompanied by characteristic patterns of patchy necrosis and disrupted myofiber alignment. Local AD-MSC administration demonstrated superior therapeutic efficacy, reducing fibrosis by 65.8% relative to the model group (7.2 ± 0.5%; *p* < 0.05), with near-complete restoration of myocardial microstructure. While systemic AD-MSC delivery achieved a 42.4% reduction in collagen deposition (12.12 ± 0.6%), this improvement did not reach statistical significance versus untreated periodontitis controls (*p* > 0.05), revealing clear therapeutic stratification between delivery modalities.

### 2.6. Conventional Echocardiographic Findings

**Figure 10:**
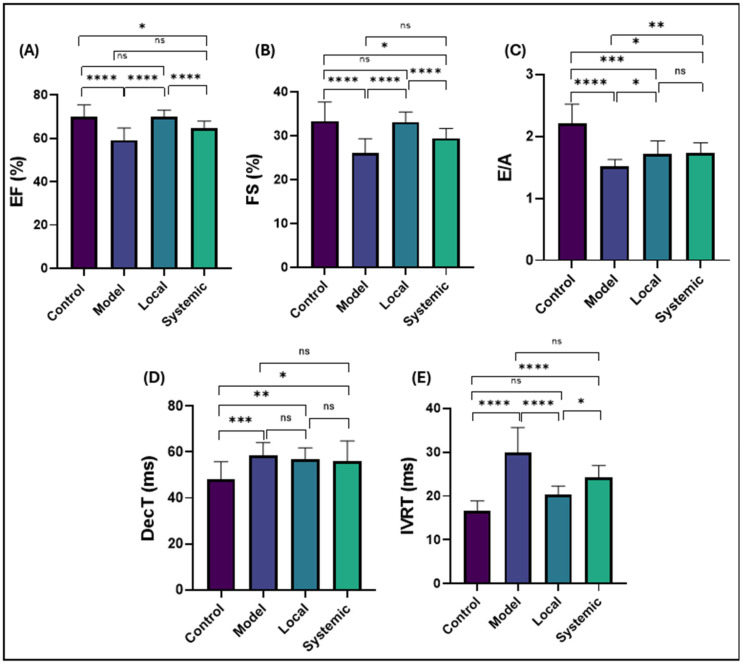
Conventional echocardiographic analysis in the experimental groups. (**A**) Ejection fraction (EF%), (**B**) fractional shortening (FS%), (**C**) E/A ratio, (**D**) deceleration time (DecT), and (**E**) isovolumic relaxation time (IVRT). Data are presented as mean ± SD. Statistical significance is indicated as follows: * *p* < 0.05, ** *p* < 0.01, *** *p* < 0.001, **** *p* < 0.0001, ns = not significant. Comparisons are shown between the control, model, local AD-MSC-treated, and systemic AD-MSC-treated groups.

Conventional echocardiographic analysis demonstrated significant differences in systolic and diastolic cardiac function among the experimental groups (Figure 10).

For systolic function, the ejection fraction (EF%) and fractional shortening (FS%) were markedly reduced in the model group compared to the control group (*p* < 0.0001 for both), indicating impairment of systolic function. Local administration of AD-MSCs significantly improved EF% and FS% compared to the model group (*p* < 0.0001) and showed no significant difference from the control group (*p* > 0.05). In contrast, systemic administration of AD-MSCs resulted in a slight improvement in EF% and FS% compared to the control group (*p* < 0.05) but remained not significantly different from the model group (*p* > 0.05).

For diastolic function, the E/A ratio was significantly reduced in the model group compared to the control group (*p* < 0.0001), reflecting impaired ventricular filling. Local AD-MSC treatment showed a significant reduction in the E/A ratio compared to the control group (*p* < 0.001) but a significant improvement compared to the model group (*p* < 0.05). Similarly, systemic AD-MSC treatment resulted in values significantly higher than the model group (*p* < 0.01) but still lower than the control group (*p* < 0.05). The deceleration time (DecT) was significantly prolonged in the model group compared to the control group (*p* < 0.001), indicating increased ventricular stiffness. In the treatment groups, DecT showed a trend toward improvement but remained significantly prolonged. Local AD-MSC treatment still exhibited significantly prolonged DecT compared to the control group (*p* < 0.01) and no significant difference from the model group (*p* > 0.05). Similarly, systemic AD-MSC treatment showed significantly prolonged DecT compared to the control group (*p* < 0.05) with no significant difference from the model group (*p* > 0.05).

The isovolumic relaxation time (IVRT) was significantly prolonged in the model group compared to the control group (*p* < 0.0001), indicating impaired myocardial relaxation. Local AD-MSC treatment significantly shortened IVRT compared to the model group (*p* < 0.0001), aligning closely with the control group (*p* > 0.05). However, systemic AD-MSC treatment resulted in IVRT values that remained significantly prolonged compared to the control group (*p* < 0.0001) and showed no significant difference from the model group (*p* > 0.05).

## 3. Discussion

This study introduces a novel comparative analysis of local versus systemic AD-MSCs’ administration in addressing both periodontal tissue damage and its systemic cardiac complications in a rat model of experimental periodontitis. While the regenerative potential of MSCs in periodontitis has been explored, the systemic cardioprotective benefits of AD-MSCs and their differential efficacy based on delivery routes remain underexamined. Our findings demonstrate that localized AD-MSC therapy not only enhances periodontal bone regeneration and reduces inflammation, but also significantly ameliorates periodontitis-induced cardiac dysfunction, outperforming systemic delivery. This dual therapeutic impact underscores the importance of targeted MSC administration in mitigating localized tissue damage while concurrently addressing systemic inflammatory cascades linked to cardiovascular disease. By bridging the gap between oral and systemic health, this work provides critical insights into optimizing MSC-based strategies for comprehensive patient care, emphasizing the clinical relevance of delivery route selection in regenerative medicine.

The fibroblast-like morphology observed in passage 4 AD-MSC cultures aligns with prior studies describing the characteristic spindle-shaped, elongated structures of MSCs. These cells adhere to plastic culture surfaces, a widely recognized trait that supports their mesenchymal origin [32,33]. Flow cytometry analysis confirmed the mesenchymal identity of AD-MSCs through the expression of CD29 and CD90, with the absence of the hematopoietic marker CD45. These findings comply with the International Society for Cellular Therapy (ISCT) criteria, which specify that MSCs must express mesenchymal markers like CD29 and CD90 while lacking hematopoietic markers such as CD45 [34,35]. CD29, an integrin family member, facilitates cell–extracellular matrix adhesion, while CD90 is essential for cell adhesion, signaling, migration, and differentiation. The absence of CD45 eliminates the possibility of hematopoietic contamination, ensuring the purity of the isolated AD-MSC population [35,36,37,38]. These results are consistent with previous reports on rat MSCs, reaffirming the reliability of these markers for MSC characterization [39,40].

The successful differentiation of AD-MSCs into adipogenic, chondrogenic, and osteogenic lineages highlights their multilineage potential, a hallmark of MSCs. These observations are consistent with studies demonstrating trilineage differentiation as a key criterion for MSC characterization and therapeutic applicability [34,35]. Furthermore, the differentiation capacity observed in rat-derived AD-MSCs aligns with earlier findings, supporting their regenerative potential [41,42]. Importantly, the preservation of this potential in passage 4 cells suggests that the employed culture conditions effectively maintain their stemness and functional properties [43].

Adipose tissue was chosen as the MSC source due to its high cell yield, minimally invasive harvest, and potent regenerative properties. AD-MSCs exhibit greater proliferative rates and trophic factor secretion (e.g., HGF, VEGF) compared to other MSC types, which may amplify their therapeutic effects in inflammatory and ischemic environments [41,44,45,46]. Their clinical accessibility and scalability further justify their selection for this study, particularly in addressing periodontitis-associated systemic complications.

The remarkable regenerative potential of MSCs extends beyond their differentiation capacity to include robust paracrine signaling through extracellular vesicles (EVs) and immunomodulatory factors. Recent studies demonstrate that MSC-derived EVs contain protective miRNAs (e.g., miR-21-5p, miR-133a-3p) that attenuate apoptosis in hypoxic cardiomyocytes by modulating CASP3 and BAX expression [47], which aligns with our observed reduction in cardiac fibrosis post-AD-MSC therapy. Furthermore, the successful reprogramming of gingival MSCs into pluripotent stem cells underscores the versatility of oral tissue-derived MSCs—a property shared by our AD-MSCs, which exhibited trilineage differentiation and CD29/CD90 positivity [48]. Notably, the anti-inflammatory (IL-6/CCL2 suppression) and pro-angiogenic (VEGF upregulation) effects of MSC-EVs reported in hypoxia models mirror our findings of improved cardiac function and periodontal regeneration, suggesting conserved therapeutic mechanisms across MSC types. These parallels highlight AD-MSCs as a strategically chosen source for addressing periodontitis-associated systemic inflammation.

Radiographic and clinical assessments revealed the superior efficacy of local AD-MSC administration in promoting periodontal regeneration, as evidenced by a significant reduction in the CEJ-to-alveolar bone crest distance and improved bone support approaching control levels. These findings align with studies highlighting the dual role of MSCs in reducing inflammation and directly contributing to tissue regeneration [22,49]. Local AD-MSC treatment outperformed systemic administration, likely due to better retention and targeted action at the defect site, consistent with Gao et al.’s findings on enhanced bone regeneration with local MSC delivery [50]. The therapeutic potential of MSCs is further supported by their secretion of paracrine factors, which promote angiogenesis, immunomodulation, and tissue repair [51,52,53,54,55]. In contrast, systemic administration showed limited efficacy in bone regeneration, potentially due to challenges with cell homing and retention at the defect site [56,57,58,59,60].

Our radiographic assessment of alveolar bone status revealed significant bone loss in the periodontitis group, while both local and systemic administration of AD-MSCs effectively preserved bone support, with local delivery demonstrating superior efficacy. These findings align with previous studies highlighting the regenerative potential of MSCs in periodontal tissue. For instance, Wang et al. reported that adipose-derived MSC transplantation improved bone mineral density and reduced alveolar bone loss through osteoinductive and anti-inflammatory mechanisms [61], while Kawaguchi et al. showed enhanced periodontal regeneration, particularly in cementum and alveolar bone, following local autotransplantation of bone marrow-derived MSCs with atelocollagen in canine models [62]. Our results further support this evidence, demonstrating marked preservation of bone structure following MSC therapy in a rat model of periodontitis. Notably, local administration of AD-MSCs nearly restored bone levels to those of healthy controls, outperforming systemic delivery. This is consistent with Gao et al., who observed improved bone regeneration with locally injected umbilical cord MSCs in a rat periodontitis model, attributed to enhanced cell retention and paracrine effects [50]. Similarly, Du et al. found significant bone preservation with local bone marrow MSC transplantation [49]. The superior outcomes of local delivery may be due to direct MSC integration into periodontal defects, whereas systemic administration is often limited by pulmonary entrapment, which reduces cell homing to target tissues [63].

Histological findings from H&E and Masson’s trichrome staining revealed distinct differences in periodontal tissue integrity among the study groups. The control group displayed well-preserved architecture, serving as a benchmark for healthy tissue, while the model group showed extensive pathological changes, including apical migration of the junctional epithelium, disrupted collagen fibers, significant alveolar bone loss, and inflammatory infiltration. These observations are consistent with the destructive nature of periodontitis, which compromises critical periodontal support structures such as the alveolar bone and periodontal ligament [64,65]. Effective treatment aims to halt this damage and promote regeneration [66,67].

Local AD-MSC treatment demonstrated superior periodontal tissue regeneration, significantly improving tissue architecture compared to the model group, consistent with earlier studies [68,69]. While most research on AD-MSCs focuses on surgical models, limited studies have investigated their effects in nonsurgical contexts. For instance, Lemaitre et al. reported findings on the formation of new periodontal tissues in comparable models [70]. The regenerative effects of AD-MSCs are primarily mediated through their trophic activity, including secretion of bioactive molecules that promote endogenous repair, angiogenesis, and anti-inflammatory effects [71,72,73]. In comparison, systemic AD-MSC administration showed moderate improvements, such as partial restoration of tissue structure and reduced inflammation, though with less organized collagen fibers and ongoing ligament remodeling. This reduced efficacy may stem from the lower concentration of MSCs reaching the defect site, highlighting the therapeutic advantage of localized delivery [21,74,75,76].

Masson’s trichrome staining revealed that local AD-MSCs restored collagen density in the periodontal ligament to 70.82 ± 2.22%, which was comparable to the control group (79.86 ± 2.27%), while systemic treatment resulted in only partial recovery (60.29 ± 1.30%). These findings are consistent with those of Mohammed et al., who reported a 65% collagen recovery in rat periodontitis following local adipose-derived MSC exosome therapy [68]. The disorganized collagen fibers observed in the systemic group align with the observations of Lu et al., who noted limited periodontal regeneration following intravenous MSC administration due to reduced tropism [22]. Overall, our results highlight the importance of localized MSC delivery for effective extracellular matrix remodeling, likely mediated by trophic factors such as TGF-β and VEGF [71].

The histopathological assessment of cardiac tissues highlights the therapeutic potential of AD-MSCs in mitigating myocardial damage linked to periodontitis. The control group exhibited normal cardiac morphology, with well-aligned cardiomyocytes, centrally located nuclei, and no signs of inflammation or necrosis, consistent with the histological features of a healthy myocardium [77,78]. In contrast, the model group showed significant pathological changes, including inflammatory cell infiltration and compromised tissue integrity, reflecting the systemic impact of periodontal disease. These may be attributed to elevated levels of serum cytokines and CRP, along with mechanisms such as bacterial dissemination and immune complex spread, as previously reported by other studies [79,80,81,82].

Local AD-MSC administration demonstrated notable improvements in cardiac tissue architecture, with reduced inflammation, partial restoration of cardiomyocyte alignment, and minimal collagen deposition. These findings underscore the regenerative and anti-inflammatory properties of MSCs, which secrete bioactive molecules and modulate immune responses, effectively reducing systemic inflammation at its source [83,84,85]. Local AD-MSC treatment reduced myocardial fibrosis by 65.8% (7.2 ± 0.5% vs. 21.04 ± 1.1% in the model group, *p* < 0.05), whereas systemic delivery achieved a 42.4% reduction (12.12 ± 0.6%, *p* > 0.05). This aligns with Gallet et al., who reported a 60% decrease in fibrosis post-MSC therapy in porcine myocardial infarction models, linked to exosome-mediated anti-inflammatory effects [86]. By directly targeting the periodontal defect, local AD-MSC treatment appears to prevent the release of inflammatory mediators associated with cardiac dysfunction, offering superior cardioprotective effects compared to systemic delivery. Although systemic AD-MSC administration also improved cardiac morphology, it resulted in pronounced collagen deposition and less effective resolution of inflammation, likely due to reduced homing efficiency and retention at the cardiac site [63,87].

To comprehensively evaluate cardiac function, we measured ejection fraction (EF%) and fractional shortening (FS%), which are gold-standard indices of systolic function reflecting ventricular contractility [88]. We also assessed the E/A ratio, deceleration time (DecT), and isovolumic relaxation time (IVRT), which are validated markers of diastolic dysfunction in rodent models [89,90]. Abnormalities in these parameters indicate impaired ventricular filling, a hallmark of periodontitis-associated cardiac injury [91,92].

The echocardiographic findings of this study emphasize the systemic impact of periodontitis on cardiac function and the therapeutic potential of AD-MSCs in mitigating these effects. In the model group, significant impairments in both systolic and diastolic functions were observed, likely driven by systemic inflammation and oxidative stress associated with periodontitis [91,92,93]. Chronic inflammation contributes to myocardial dysfunction through mechanisms such as cytokine-induced cardiomyocyte apoptosis, extracellular matrix remodeling, and microvascular dysfunction [94]. Moreover, several studies have established a clear association between the severity of periodontitis and cardiac dysfunction in human patients, further supporting this connection [95,96,97].

The local administration of AD-MSCs significantly enhanced cardiac function, as evidenced by EF% and FS% values approaching those of the control group. These findings are consistent with previous research highlighting the cardioprotective effects of MSCs, primarily attributed to their paracrine secretion of anti-inflammatory and pro-regenerative factors [86,98]. These mechanisms likely mitigated the systemic inflammatory burden and promoted myocardial repair, resulting in improved ventricular function. Additionally, the normalization of IVRT in the local treatment group indicates superior myocardial relaxation, reflecting reduced fibrosis and improved cardiomyocyte alignment [19]. This aligns with histological findings of decreased collagen deposition and restored myocardial architecture in this treated group. These findings can be attributed to the paracrine activity of MSCs, including the secretion of anti-inflammatory cytokines, growth factors such as VEGF, and other regenerative molecules, which play a central role in cardiac protection [87]. Local delivery ensures a sustained anti-inflammatory and reparative environment at the periodontal site (injury site), effectively ameliorating the propagation of systemic inflammation to the myocardium. This mechanism aligns with previous studies demonstrating that MSC therapies significantly reduce systemic oxidative stress and promote myocardial repair [99].

Systemic administration of AD-MSCs improved cardiac function, though less effectively than local delivery. This reduced efficacy may stem from challenges including poor cell homing and limited retention at the cardiac site [100,101]. Fischer et al. reported that a significant proportion of intravenously administered MSCs are sequestered in non-target organs, such as the lungs, limiting their therapeutic impact on the myocardium [63]. Similarly, systemic delivery poses risks of vascular entrapment and reduced targeting efficiency, as observed in other studies [102,103]. These findings highlight the need for optimizing systemic MSC delivery methods to improve therapeutic outcomes.

The persistent abnormalities in the DecT and trans-mitral E/A ratio observed across all treatment groups emphasize that, while AD-MSC therapy can mitigate certain aspects of myocardial dysfunction, achieving full restoration of diastolic function remains a challenging objective. These findings highlight the need for further optimization of MSC therapy, particularly strategies aimed at improving cell homing, survival, and functional integration within the myocardial environment.

Diastolic dysfunction is often more resistant to recovery than systolic dysfunction due to the chronic nature of myocardial remodeling and fibrosis [104]. The more rapid improvement in systolic function compared to diastolic function in our study can be attributed to the immediate effects of MSC therapy on reducing inflammation in the cardiac tissue, which reflects on myocardial inotropy, with partial reversal of the existing fibrosis. Systolic recovery is primarily driven by MSC-mediated paracrine effects that enhance cardiomyocyte function and contribute to structural repair [105,106,107,108]. In contrast, the recovery of diastolic function involves more intricate mechanisms, including extracellular matrix remodeling, calcium-handling recovery, and normalization of ventricular compliance, which require extended timeframes for complete restoration [109,110,111]. The persistent abnormalities in diastolic parameters, such as E/A and DecT, observed in this study suggest ongoing ventricular remodeling despite significant reductions in fibrosis [112]. Local administration of AD-MSCs appears to provide a more sustained reduction in fibrosis at its source, which may indirectly contribute to cardiac improvement. This is supported by histological evidence demonstrating significantly reduced myocardial collagen deposition in the local treatment group compared to the systemic delivery group. The superior efficacy of local MSC therapy highlights the critical role of targeting systemic inflammation at its origin, thereby promoting more comprehensive cardiac repair.

### Clinical Significance of Findings

Our study demonstrates that local administration of AD-MSCs not only enhances periodontal regeneration but also mitigates periodontitis-associated cardiac dysfunction, offering a dual therapeutic strategy for a disease with systemic health implications. Clinically, these findings support the use of minimally invasive, targeted MSC therapies as an adjunct to conventional periodontal treatments (e.g., scaling/root planing or guided tissue regeneration), particularly in patients with pre-existing cardiovascular risks. The superior efficacy of local delivery suggests that direct application at the defect site maximizes MSC retention and paracrine effects, aligning with recent trials using dental pulp stem cells for intrabony defects [29]. Moreover, the observed improvement in cardiac systolic function underscores the potential of periodontal therapy to reduce systemic inflammation-driven cardiovascular damage. This is critical given the established link between periodontitis and atherosclerosis [5] or atrial fibrillation [10]. Our rat model provides a translational foundation for human trials combining periodontal and cardioprotective MSC therapies, especially in high-risk cohorts (e.g., diabetics or elderly patients).

## 4. Materials and Methods

### 4.1. Animal Care and Ethical Compliance

This study utilized 24 healthy male Sprague-Dawley rats (8 weeks old, 300–350 g) housed under controlled conditions, including a temperature of 23 °C, a 12 h light/dark cycle, and 40–60% humidity. The rats had unrestricted access to food and water. All procedures adhered to the guidelines of the Animal Care and Use Committee of Tokyo University of Agriculture and Technology (approval number: R05-159). Euthanasia was performed humanely via isoflurane overdose inhalation at the end of the experiment.

### 4.2. Preparation and Culturing of AD-MSCs

Adipose tissue from rat inguinal fat was washed with phosphate-buffered saline (PBS) (cat. no. 09-8912-100, Medicago AB, Uppsala, Sweden), minced, and digested with 0.1% collagenase type I (cat. no. 17100017, Gibco by Life Technologies, Waltham, MA, USA) in HBSS (cat. no. 14025-092, Thermo Fisher Scientific Inc., New York, NY, USA) for one hour at 37 °C. The enzyme activity was neutralized with cold HBSS, and the mixture was centrifuged to obtain a cell pellet. The pellet was then filtered using a 100 μm filter (BD Falcon, Bedford, MA, USA) to remove aggregates, and red blood cells were lysed. The resulting pellet was resuspended in DMEM (cat. no. 043-30085, FUJIFILM Wako Pure Chemical Corporation, Osaka, Japan) supplemented with 10% FBS (cat. no. CCP-FBS-BR-500, COSMO BIO, Tokyo, Japan), non-essential amino acids (cat. no. 139-15651, FUJIFILM Wako Pure Chemical Corporation, Osaka, Japan), and penicillin-streptomycin (cat. no. 161-23181, FUJIFILM Wako Pure Chemical Corporation, Osaka, Japan).

Cells were cultured in 100 mm dishes at 37 °C with 5% CO_2_, with medium changes every three days until they reached 80% confluence. They were then trypsinized, subcultured, and expanded to passage four, at which point they were examined microscopically (Olympus CKX31, Tokyo, Japan) and prepared for further analysis.

### 4.3. Flow Cytometric Characterization of AD-MSCs

Flow cytometry was employed to confirm the immunophenotype of AD-MSCs. The MSC-specific markers CD29 (PE, eBioscience™, San Diego, CA, USA, Catalog # 12-0291-82) and CD90 (PE, eBioscience™, Catalog # 12-0900-81), and the hematopoietic marker CD45 (PE, Catalog # MA5-17379) were detected. Isotype controls included Armenian Hamster IgG (PE, eBioscience™, Catalog # 12-4888-81), Mouse IgG2a kappa (PE, eBioscience™, Catalog # 12-4724-42), and Mouse IgG2a (PE, Catalog # MG2A04) [113,114].

Cells were washed with HBSS, resuspended in FACS buffer (PBS supplemented with 2% fetal bovine serum and 0.1% sodium azide), and adjusted to a concentration of 1 × 10^6^ cells/mL. The cell suspension was then incubated with antibodies (CD29-PE, CD90-PE, CD45-PE) at manufacturer-recommended concentrations for 20 min at 4 °C in the dark. After washing to remove unbound antibodies, surface antigen expression was analyzed using a Beckman Coulter flow cytometer (Beckman Coulter, Brea, CA, USA). Data processing and analysis were conducted using CytExpert Software version 2.3 (Beckman Coulter, Brea, CA, USA).

### 4.4. Multilineage Differentiation Assessment of AD-MSCs

To assess the differentiation potential of passage-four AD-MSCs, the cells were induced to differentiate into adipocytes, osteocytes, and chondrocytes, while control cells were cultured in standard growth medium (DMEM supplemented with 10% FBS).

#### 4.4.1. Adipogenic Differentiation

AD-MSCs (passage 4) were plated in a 6-well plate at a density of 1 × 10^5^ cells per well. Once the cells reached 80–100% confluence, adipogenic differentiation was initiated using an induction medium. This medium consisted of DMEM supplemented with 10% FBS, 1 μM dexamethasone (Cat. no. D4902, Sigma Aldrich, St. Louis, MO, USA), 500 μM isobutylmethylxanthine (Cat. no. I5879, Sigma Aldrich, St. Louis, MO, USA), 100 μM indomethacin (Cat. no. I7378, Sigma Aldrich, St. Louis, MO, USA), and 5 μg/mL insulin (Cat. no. I5500, Sigma Aldrich, St. Louis, MO, USA). A standard culture medium containing DMEM and 10% FBS was used as a negative control. The medium was refreshed every three days for all wells. After 21 days, the cells were collected and stained with Oil Red O (Cat. no. O-0625, Sigma-Aldrich, St. Louis, MO, USA) to detect intracellular lipid accumulation. The formation of red-stained lipid droplets confirmed successful adipogenic differentiation.

#### 4.4.2. Chondrogenic Differentiation

AD-MSCs at passage four were plated in a 6-well plate at a density of 1 × 10^5^ cells per well using a serum-free medium specifically formulated for chondrogenic differentiation (Cat. no. C-28012, PromoCell GmbH, Heidelberg, Germany). The medium was refreshed every three days for a total of 21 days. Chondrogenic differentiation was assessed through Alcian Blue staining (Cat. no. 66011, Sigma-Aldrich, St. Louis, MO, USA), which selectively binds to highly sulfated proteoglycans in the cartilaginous matrix. The stained samples were then examined under a light microscope to confirm chondrogenesis.

#### 4.4.3. Osteogenic Differentiation

AD-MSCs at passage four were plated in a 6-well plate at a density of 1 × 10^5^ cells per well. Once they reached 80–100% confluence, osteogenic differentiation was induced using a specialized medium containing DMEM supplemented with 10% FBS, 100 nM dexamethasone, 0.2 mM ascorbic acid (Cat. no. 016-04805, FUJIFILM Wako Pure Chemical Corporation, Osaka, Japan), and 10 mM β-glycerophosphate (Cat. no. G9422, Sigma Aldrich, St. Louis, MO, USA). Control wells were maintained in an uninduced culture medium. The media were replaced every three days for a total of 21 days. Osteogenic differentiation was evaluated using Alizarin Red staining (ALZ) (Cat. no. 40-1009-5, Sigma-Aldrich, St. Louis, MO, USA), which binds to the mineralized matrix. The presence of red-stained deposits confirmed successful osteogenic differentiation.

### 4.5. Experimental Groups and Treatment Protocols

Rats were randomly divided into four groups (*n* = 6 per group):

1. Control Group: No ligation or AD-MSC treatment.
2. Model Group: Ligation-induced periodontitis without AD-MSC treatment.
3. Local Group: Ligation followed by local AD-MSC injections (1 × 10^6^ cells) delivered supraperiosteally near the bone surface.
4. Systemic Group: Ligation followed by systemic AD-MSC injections (1 × 10^6^ cells) via the tail vein.

Anesthesia was induced using medetomidine hydrochloride (0.3 mg/kg), midazolam (5.0 mg/kg), and butorphanol (5.0 mg/kg), administered subcutaneously. Atipamezole (1.0 mg/kg) was used for recovery [115,116].

**Figure 11:**
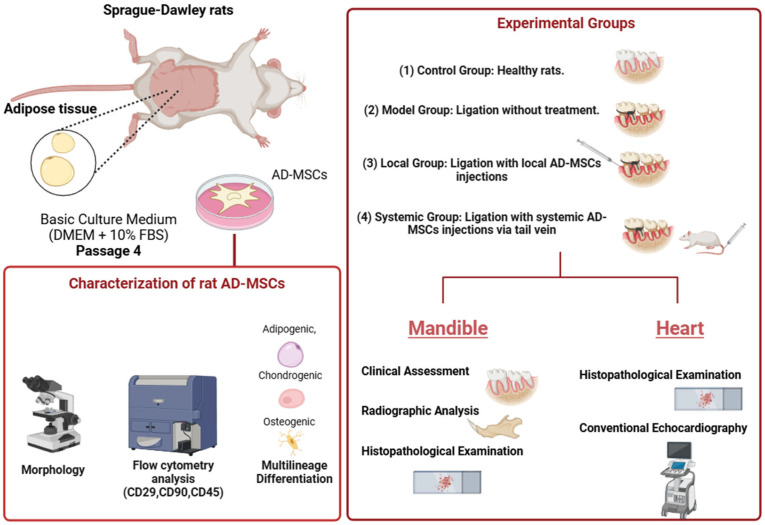
Overview of the experimental design.

Ligation was performed on day 0 by placing sterile silk sutures (USP size 3-0) around the mandibular first molars to induce periodontitis as prescribed in our previous study [92]. Ligatures remained for 60 days and were then removed. AD-MSCs (1 × 10^6^ cells suspended in PBS) were administered weekly for three weeks starting from ligature removal [117]. All rats were sacrificed on day 88, and mandibular and cardiac tissues were collected for analysis. The experimental design is shown in Figure 11.

### 4.6. Clinical Assessment of Periodontal Bone Loss

Following euthanasia, the hemimandibles were carefully isolated and dissected. After thorough cleaning with PBS, the specimens were photographed using a stereomicroscope (Leica M60) (Leica M60, Leica Microsystems, Wetzlar, Germany). Clinical bone loss was quantified following the method described by Oliveira et al. [118] using ImageJ software (version 1.8.0-345), available at https://imagej.nih.gov/ij/download.html (accessed on 25 December 2024). The measurement process involved determining the linear distance from the cemento-enamel junction (CEJ) to the alveolar bone crest in millimeters, with the final value reported as the average of three independent measurements.

### 4.7. Radiographic Assessment of Alveolar Bone Loss and Periodontal Bone Support

Radiographic evaluations were performed using an X-ray machine Shimadzu, (Collimator Type R-20J, Shimadzu Corporation, Kyoto, Japan). The X-ray tube functioned at 30 kW with a current of 6 mA for an exposure time of 0.01 s, while the distance between the radiation source and the sensor was maintained at 50 cm. Alveolar bone loss was evaluated radiographically following the methodology outlined by Holzhausen et al. [119]. The distance between the cemento-enamel junction (CEJ) and the alveolar bone crest was measured at the mesial root surfaces of the mandibular first molars in the affected area using ImageJ software (version 1.8.0-345).

Periodontal bone support was assessed based on the approach described by Andersen et al. [120] and Shirakashi et al. [121]. Digital measurements were performed on the mesial aspect of the mandibular first molars. A reference line (AC) was established from the cusps (C) of the first molar to the apex (A) of its corresponding root. Another line (AB) was drawn from the apex (A) to the level of the deepest interproximal bone defect (B). The measurements, recorded in millimeters, were used to calculate periodontal bone support using the formula:

Periodontal Bone Support = (AB/AC) × 100%.

### 4.8. Histopathological Examination

Hematoxylin and Eosin (H&E) staining and Masson’s trichrome staining were performed to assess structural alterations, inflammatory responses, and fibrotic regions in the mandibular and cardiac tissues.

#### 4.8.1. Mandibular Tissues

Following euthanasia via isoflurane overdose (5% inhalation), mandibular specimens were immediately collected and fixed in 10% neutral buffered formalin for 48 h. After fixation, the tissues were decalcified in 8% nitric acid for seven days to facilitate proper sectioning. Post-decalcification, the specimens were dehydrated in a graded series of methanol, cleared in xylene, and embedded in paraffin. Paraffin-embedded tissue blocks were sectioned to a thickness of 4 μm, mounted on glass slides, and air-dried for two hours. The sections were then deparaffinized in xylene, rehydrated through graded ethanol, and rinsed in running water before staining. Mandibular specimens were sectioned in the mesiodistal plane to include the first molar and its periodontal structures. Histopathological assessments qualitatively evaluated the apical migration of the junctional epithelium and the organization of the periodontal ligament (PDL).

#### 4.8.2. Cardiac Tissues

Cardiac tissues were collected during necropsy and dissected into smaller fragments, considering the cutting aspect (transverse). The tissue was fixed in 10% neutral buffered formalin at room temperature for 48 h. Similar to the protocol for mandibular tissues, the cardiac specimens were dehydrated in methanol, cleared in xylene, and embedded in paraffin. The paraffin-embedded sections were cut to a thickness of 4 μm, mounted on slides, and air-dried for two hours. Deparaffinization, rehydration, and rinsing were performed as previously described for the mandibular tissues.

#### 4.8.3. Staining Procedures

##### H&E Staining

H&E staining was carried out using Carazzi’s Hematoxylin (Catalog no. 3002-2, MUTO PURE CHEMICALS Co., Ltd., Tokyo, Japan) and 1% Eosin Y solution (Catalog no. 051-06515, FUJIFILM Wako Pure Chemical Corporation, Osaka, Japan). Histological assessments were conducted by two independent, blinded observers, who analyzed 10 sections per experimental group. The analysis focused on identifying mononuclear cell infiltration, interstitial edema, necrosis, and the organization and alignment of myocardial cells. Observations were performed using a digital imaging microscope (BZ-9000, KEYENCE, Osaka, Japan).

##### Masson’s Trichrome Staining

To evaluate interstitial fibrotic regions, Masson’s trichrome staining was performed on both mandibular and cardiac tissues using the Trichrome Stain Kit (Connective Tissue Stain) (Catalog No. ab150686, Abcam, Tokyo, Japan), following the manufacturer’s instructions. This staining allowed for the visualization of collagen deposition and fibrotic changes in the tissues.

Collagen density was measured in Masson’s trichrome-stained sections using ImageJ 1.53v. For each sample (*n* = 6/group), three 40× fields were analyzed by isolating blue collagen staining via color deconvolution (Masson Trichrome preset), applying auto-thresholding, and calculating the percentage of stained area relative to total tissue area. This protocol was applied consistently to both periodontal ligament and cardiac tissue analyses.

### 4.9. Conventional Echocardiography

Cardiac function was assessed in all experimental groups one day before euthanasia using a ProSound 7 ultrasonographic system (Hitachi-Aloka Medical Ltd., Tokyo, Japan) equipped with a 12 MHz transducer supported by Continuous Motion Mode Echocardiography (CMME) and simultaneous electrocardiography (ECG). Echocardiographic evaluations were performed following the guidelines of the American Society of Echocardiography (ASE) [122,123].

For left ventricular (LV) assessment, a two-dimensional right parasternal short-axis view at the level of the papillary muscles was acquired, followed by M-mode echocardiography. The parameters measured included ejection fraction (EF%) and fractional shortening (FS%), which were calculated as averages from at least five consecutive cardiac cycles on M-mode tracings to ensure accuracy.

Additionally, diastolic function was evaluated using pulsed-wave Doppler (PW Doppler) echocardiography. From the left apical four-chamber view, trans-mitral inflow indices, including the E/A ratio (the ratio of early (E) to late (A) ventricular filling velocities), were recorded. Supporting diastolic parameters, such as deceleration time (DecT) and isovolumic relaxation time (IVRT), were also measured. All Doppler values were derived as averages of five consecutive cardiac cycles for consistency and reliability.

### 4.10. Statistical Analysis

Statistical analyses were performed using GraphPad Prism 8.0 (GraphPad Software, San Diego, CA, USA). Group comparisons were conducted using the Kruskal–Wallis test followed by Dunn’s post hoc test for pairwise comparisons. Data are presented as mean ± standard deviation (SD). A *p*-value of <0.05 was considered statistically significant. Graphical representations of the data were generated using the same software.

## 5. Conclusions

This study establishes the superior efficacy of locally administered AD-MSCs over systemic delivery in a rat model of experimental periodontitis, demonstrating significant improvements in periodontal regeneration and concurrent mitigation of cardiac dysfunction. These findings underscore the therapeutic advantage of localized MSC delivery, which ensures targeted retention and sustained paracrine action at injury sites while indirectly alleviating distant organ damage. This dual regenerative and systemic protective capacity positions AD-MSCs as a promising strategy for managing periodontitis and its cardiovascular sequelae.

### Limitations

The study lacks detailed molecular analyses to elucidate the mechanisms underlying the therapeutic effects of AD-MSCs. Specifically, the roles of paracrine factors such as cytokines, growth factors, and exosomes in mediating tissue regeneration and reducing inflammation were not investigated. Additionally, the absence of molecular markers for angiogenesis, inflammation, and tissue remodeling limits the ability to correlate histological and functional findings with cellular and molecular pathways. Future studies with extended follow-up periods are necessary to fully elucidate the long-term impact of MSC therapy on diastolic recovery.

## Figures and Tables

**Table 1:** Alveolar bone destruction in studied rats.

	Control	Model	Local	Systemic
Bone Loss (mm)	0.59 ± 0.14	1.46 ± 0.23 ***	0.82 ± 0.069 ^ns^	1.11 ± 0.07 *
Bone Support (%)	59.51 ± 5.37	30.33 ± 2.95 ***	50.20 ± 3.02 ^ns^	45.87 ± 3.84 ^ns^

Values are presented as mean ± SD. *** *p* < 0.001 (Model vs. Control), * *p* < 0.05 (Systemic vs. Control), ns = not significant (Local vs. Control).

## Data Availability

The collected literature is available on request.
